# Insect wing 3D printing

**DOI:** 10.1038/s41598-021-98242-y

**Published:** 2021-10-14

**Authors:** Kazuya Saito, Hiroto Nagai, Kai Suto, Naoki Ogawa, Young ah Seong, Tomohiro Tachi, Ryuma Niiyama, Yoshihiro Kawahara

**Affiliations:** 1 Faculty of Design, Kyushu University, Fukuoka, 815-8540 Japan; 2 Graduate School of Engineering, Nagasaki University, Nagasaki, 852-8521 Japan; 3 Graduate School of Arts and Sciences, The University of Tokyo, Tokyo, 153-8902 Japan; 4 Tokyo University of Agriculture, Kanagawa, 243-0034 Japan; 5 Graduate School of Information Science and Technology, The University of Tokyo, Tokyo, 113-8654 Japan; 6 Graduate School of Engineering, The University of Tokyo, Tokyo, 113-8654 Japan; 7 Nature Architects Inc., Tokyo, 107-0052 Japan

**Keywords:** Aerospace engineering, Entomology

## Abstract

Insects have acquired various types of wings over their course of evolution and have become the most successful terrestrial animals. Consequently, the essence of their excellent environmental adaptability and locomotive ability should be clarified; a simple and versatile method to artificially reproduce the complex structure and various functions of these innumerable types of wings is necessary. This study presents a simple integral forming method for an insect-wing-type composite structure by 3D printing wing frames directly onto thin films. The artificial venation generation algorithm based on the centroidal Voronoi diagram, which can be observed in the wings of dragonflies, was used to design the complex mechanical properties of artificial wings. Furthermore, we implemented two representative functions found in actual insect wings: folding and coupling. The proposed crease pattern design software developed based on a beetle hindwing enables the 3D printing of foldable wings of any shape. In coupling-type wings, the forewing and hindwing are connected to form a single large wing during flight; these wings can be stored compactly by disconnecting and stacking them like cicada wings.

## Introduction

Insects are the most prosperous group of terrestrial animals and have spread into nearly all environments on this planet. Discovering the essence of their excellent environmental adaptability and locomotive ability can help broaden the human movement capabilities through the development of innovative mobility technology. Therefore, researchers have attempted to imitate various methods of insect locomotion, including walking^1–3^, crawling^4–6^, and jumping^7–9^. Among them, the excellent flight ability of insects has been drawing particular attention^10–24^, which is represented by the incredible maneuverability observed in dragonflies^17^ or flies^15,25^ and the energy-efficient flight of butterflies during overseas migration^10,11^. Researchers have proposed various types of flapping micro air vehicles (MAVs)^25–30^; however, the abilities of MAVs are limited compared to those of real insects. A major problem is the difficulty in reproducing complex mechanical properties and various functions in insect wings. Insect wings are sophisticated membrane structures that are supported by frames with complicated patterns^19,21–24^ that cause complex stiffness distribution and anisotropy^31^. They deform flexibly while interfering with the surrounding air during flapping, producing a useful aerodynamic effect^12–14,31^. Therefore, flapping wings are much more difficult to design and manufacture than fixed and rotary wings. Furthermore, most insect wings have flight functions as well as various other functions represented by folding^32–41^ and coupling^42,43^. These additional functions of the wings are key factors for the adaptability of insects, because they enable a combination of excellent flight ability with other locomotion methods, such as walking and crawling. However, few studies have implemented these important functions. Researchers have proposed various types of artificial wings by processing carbon-fiber composite materials^25–30^; however, these techniques require special facilities, materials, and knowledge/expertise. The difficulty of sharing this knowledge constitutes a serious obstacle to the construction of interdisciplinary research groups that are indispensable for biomimetics.

This research aims to establish a highly versatile method of implementing bioinspired designs from various types of insect wings, including complex mechanical properties and unique functions, using a simple method that anyone can easily realize, and to overcome the above-mentioned difficulty of knowledge sharing. In this study, a commercially available 3D printer was used to form a vein-like structure directly on a thin film. The proposed method enables the design of a complex distribution of stiffness and the implementation of various functions in artificial wings. In this study, we created three types of artificial wings: dragonfly, beetle, and cicada. These models cover three important functions in insect wings: complex deformation during flight, folding, and coupling. We used a fused deposition modeling (FDM)-type 3D printer with a heated bed for manufacturing. The film was fixed on a printing bed using a vacuum pump (Fig. S1). By using the same type of material for the film and filament, it is possible to confer high adhesion without peeling off after fabrication. We selected polypropylene (PP) from among the commodity plastics. PP has good material properties (high mechanical strength, heat resistance, and low specific weight) and is easily available in film shapes, such as packaging and envelopes.

## Results

**Figure 1:**
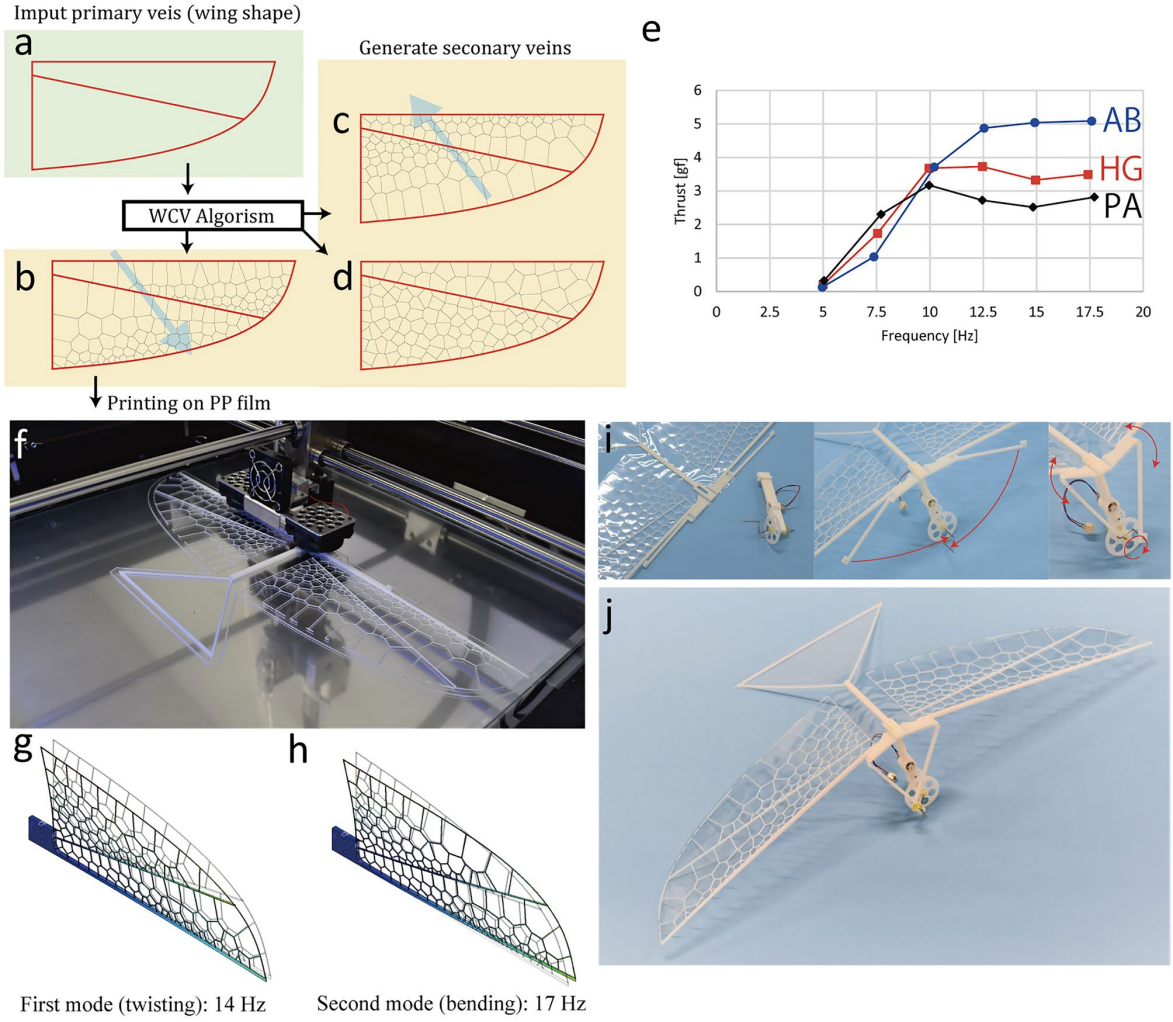
3D printed wings with artificial Voronoi venations. (**a**–**d**) Design process for wing venation. The wing shape and primary veins were manually designed. The artificial venation program generates secondary veins using WCV patterns with selected weight parameters and directions. (**e**) Thrust test results. (**f**) Wing venation printed on PP film using an FDM 3D printer. (**g**, **h**) First (twisting) and second (bending) vibration modes of the AB-type wing simulated by Fusion 360. The general properties of the PP plastic material were used. (*E* = 1.34 GPa, *ν* = 0.392, *ρ* = 899 kg/m^3^). (**i**) Flapping mechanisms built with wings. (**j**) One-piece build flapping drone (except for the motor unit).

Unlike in fixed and rotary wings, wing flexibility^13,14^ is actively used to produce a high aerodynamic effect in flapping wings. In previous studies, flapping wings were usually manufactured using carbon frames^26–28^, which were processed into shapes that imitated primary veins with the membranes. However, in actual insect wings, mesh-like secondary veins support the primary veins. Finite element analysis indicated that the primary and secondary veins affected the mechanical properties of the wing^31^. To design the bending and torsional properties of an entire wing as well as the stiffness and weight distribution of the membrane with simple FDM 3D printing, this study proposes a method that automatically generates secondary veins among the pre-designed primary veins, similar to actual insect wings. We focused on the centroidal Voronoi pattern found in the wings of dragonflies and developed an automatic generation program for artificial venation, enabling various wing shapes with partially different mechanical properties. Figure 1 shows a schematic of the proposed method. First, the wing shape and primary veins were manually designed. The primary veins determine the basic mechanical properties and can be used to implement functional structures, such as articulations and coupling, as discussed later. This study uses the weighted centroidal Voronoi (WCV) tessellation for the secondary vein pattern. The venation-generating algorithm was constructed with Rhino 6.0 (Robert McNeel & Associates) and Grasshopper^44^. The algorithm generates WCV patterns among the primary veins (Fig. 1b–d). The Lloyd algorithm was used to generate WCV patterns^45^. The centroidal Voronoi (CV) tessellation is the Voronoi diagram, where the generator and centroid of each Voronoi cell position are located at the same point. The WCV tessellation has weighted centroids, and the cells are distributed according to the weight function. The weighted centroid C_*i*_ is expressed as follows.

1 $$\frac{{\int_{A} {x\rho \left( x \right)dA} }}{\rho \left( x \right)dA}$$

Here, A is the area of the Voronoi cell, and *ρ*(*x*) is the density function defining the weight. The Lloyd method is an algorithm that determines the CV pattern from the initial generator through iterative calculations. By updating the Voronoi diagram with the centroid of the Voronoi cell as the next generator, the generator gradually converges to the centroid, and the CV pattern is obtained. The algorithm can generate homogeneous Voronoi patterns and change the distribution of the Voronoi seeds partially by controlling the weight parameter and direction, enabling the design of distributed stiffness in the wing membrane. The detailed operation of the software is explained in SM text 1 and Movie S1.

When fabricating veins with FDM, the size of the vein is limited by the nozzle diameter. It is impossible to create a vein that is smaller than the nozzle diameter. The advantage of the above method is that the complex distributed mechanical properties can be realized only by changing the geometrical parameters, such as the density of the seeds and weighting parameters, enabling the production of complex mechanical properties in the flapping wing via simple FDM.

To confirm the effect of the artificial venations, we designed three types of wings. Each wing has a simple shape, with a length of 100 mm and width of 50 mm, and a straight primary vein that crosses diagonally from the wing base to the apical posterior edge (Fig. 1a). Secondary veins were generated using the WCV algorithm with different weight directions. Figure 1b–d show the wing with the WCV weighted in the anterior basal direction (AB type), with homogeneous Voronoi without weight (HG type), and with the WCV weighted in the posterior apical direction (PA type), respectively. The obtained patterns were printed on PP films, and the wings were manufactured using the proposed method (Fig. 1f, Movie S2).

The effects of different WCV venations on the flapping flight properties were investigated via a simple flapping thrust test using these three wings (Materials and methods, Fig. S2). Figure 1e shows the results. The AB type wing was approximately 36% higher than the other two types. We also conducted modal frequency analysis using the designed 3D models and calculated the mode shapes and their natural frequencies. A 3D modeling software (Fusion 360 Autodesk Inc., San Rafael) was used with the material properties of the general PP plastic. Figure 1g and h show the vibration modes shaped in the first and second modes, respectively. Including other wing models, twisting and bending are dominant in the first and second modes, respectively. The natural frequencies of the first and second modes, respectively, are approximately 14 and 17 Hz for the AB type, 10 and 16 Hz for the HG type, and 9.5 and 16 Hz for the PA type. The AB type showed the highest natural frequency and a small difference between the first and second modes. The results show that changing the WCV pattern that control the weight and stiffness distribution in the wing can tune the natural frequencies of the bending and twisting modes, enabling to improve the aerodynamic characteristics in the printed wings.

We also developed a flapping mechanism using PP-compliant hinges that could be built with wings simultaneously (Fig. 1i, j). This technique enables the production of entire flapping drones, including the main wing, flapping mechanism, body, and tail wing, using a 3D printer (Movie S2). The 3D models of the flapping MAVs are described in Ref.^46^. The drastic simplification of the assembly process is expected to significantly reduce transportation costs due to on-site production as well as realize rapid trial and error, which are highly useful for experimental approaches.

**Figure 2:**
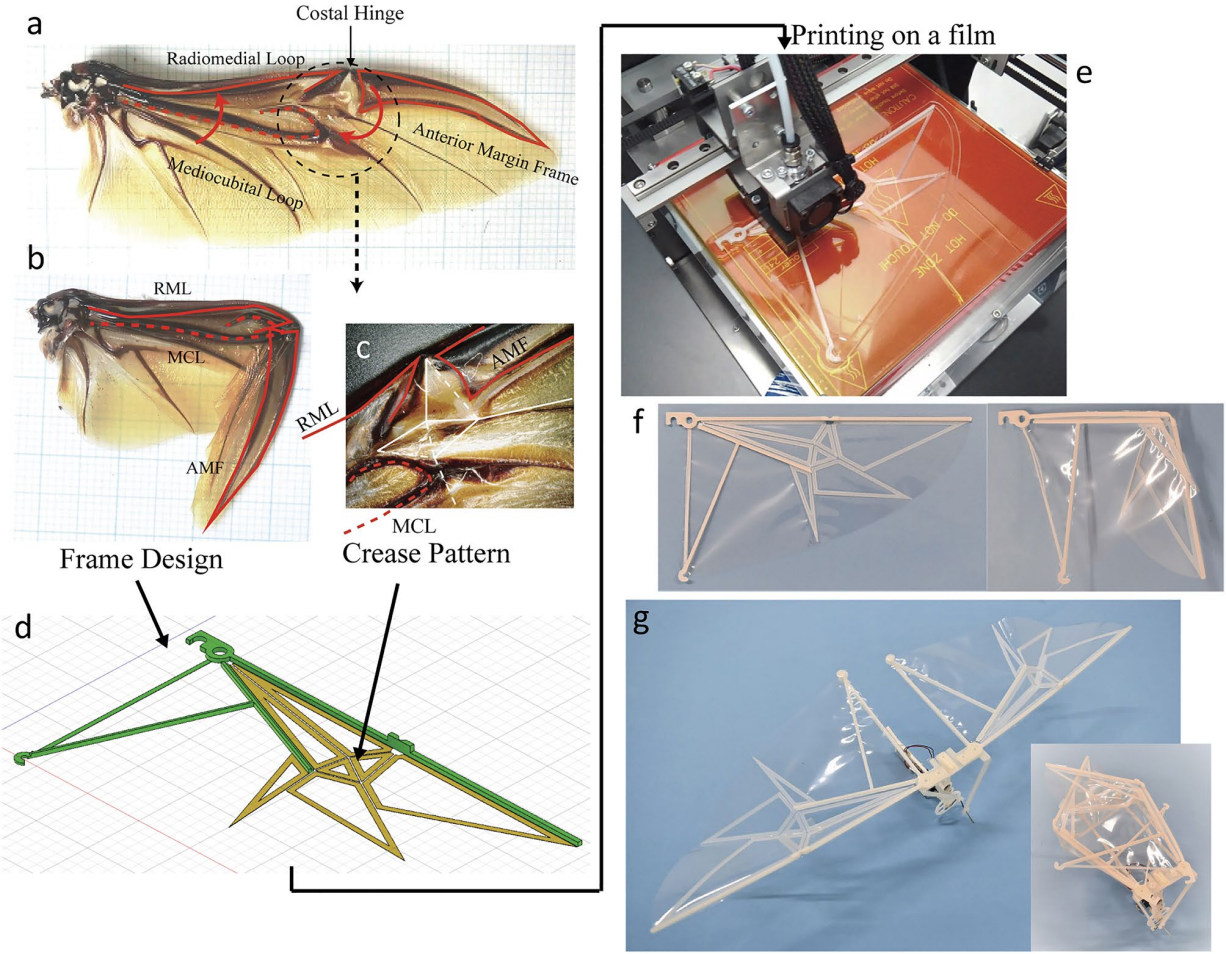
Design of the beetle inspired foldable wing. (**a**, **b**) Hindwing in a Japanese horn beetle. Wing is supported with three main frames. (**c**) Main crease pattern found at the center of the wing. (**d**) 3D model of designed foldable wing. Frames (green) are designed based on above three main frames. Crease patterns are introduced by reinforcing facets with 0.1 mm clearance (yellow). (**e**) 3D printing of wing structures on PP film. (**f**, **g**) 3D printed foldable wings and flapping drone.

Although the wings allow high-speed, long-distance travel, they significantly limit their locomotion on the ground without storage capability. Folding is a representative technique that insects use for wing storage, and it can be used to overcome the disadvantages of having wings that are brittle and bulky, enabling high-speed locomotion and access to narrow spaces. Foldable wings are complicated deployable structures and are commonly made with multiple frames and joints, which require a complex manufacturing process. Herein, we present a simple method for designing and manufacturing integrated formed foldable wings based on the wing structures and crease patterns found in large sized beetles. This wing folding can achieve compact storage and quick folding/unfolding without compromising the strength and stiffness of the wing. Figure 2a–c show the main wing frames of a Japanese horn beetle, which we referred to earlier. We note that the characteristic triangular pattern^39^ (Fig. 2c) plays an important role in wing folding. First, the crease pattern is modified according to the objective wing shape based on the flat-foldability condition. Based on this crease pattern, a wing supporting the mainframe and fold lines was designed. The green parts in Fig. 2d represent the frames with articulation, and the yellow parts represent the crease pattern. The articulation in the frame is implemented as a simple compliant hinge, using the high toughness and durability of PP, for easy production. The crease patterns were printed by reinforcing the edges of the facets with gaps on the fold lines. The proposed foldable wing can be 3D printed similar to the WVC wing (Fig. 2e). Figure 2f and g show the unfolded/folded shapes of the foldable and flapping drones. The detailed design process is presented in SM text 2 and Movie S3. The proposed method can employ different shaped frames and crease patterns and can manufacture foldable wings of various shapes without an assembly process.

Coupling-type wings are also used in various insect species, such as Hemiptera and Hymenoptera^42,43^. This type of insect uses two pairs of wings for flight; however, the posterior and anterior wings are connected by a coupling structure and flapped as a single large wing during flight (Movie S4). An important feature of the coupling wing is its high aerodynamic effect^27,42^. The coupling structure can enlarge the wing area by connecting two wings, and it can actively change the wing cross-section by moving the anterior and posterior wings around the coupling points, which can realize high aerodynamic forces. Another important function of the coupling wing is its capability to disconnect quickly after landing, such that the insect can overlap two wings and store them in a compact shape. This method is employed by several species of insect and is a suitable solution to satisfy both high flight ability and comfortable life on the ground and on plants.

**Figure 3:**
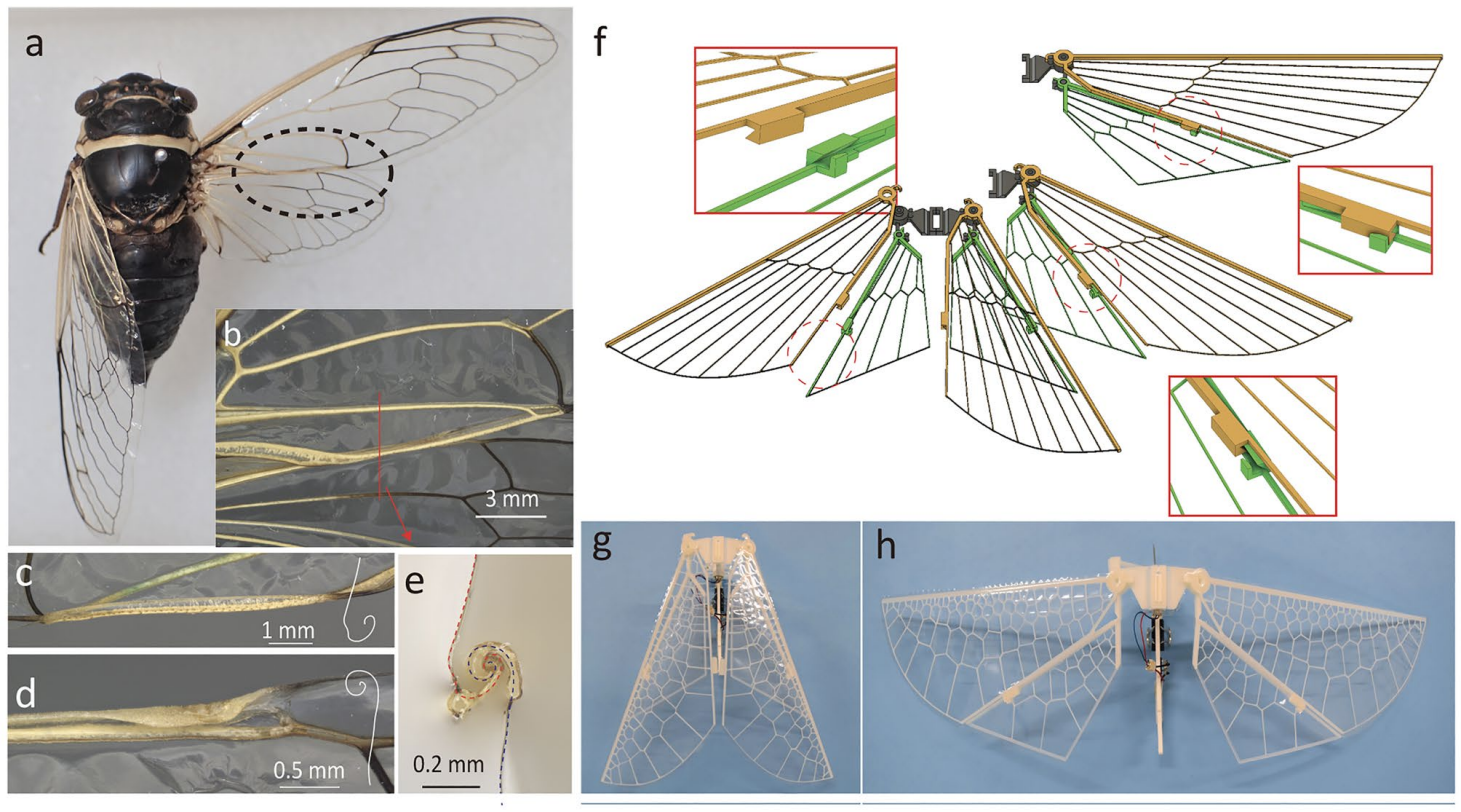
Design of coupling wing. (**a**, **b**) Wings of a cicada (*Diceroprocta apache* (Davis, 1921)). Cicadas use two pairs of wings (forewings and hindwings) for flight. The trailing edge of the forewing and the leading edge of the hindwing have coupling structures with hook-shaped cross sections, which connect the two wings during flight. The coupling structures are disconnected, and the forewings are overlapped on the hindwings for compact storing after landing on a tree. (**c**, **d**) Coupling structures on a forewing and hindwing, and schematics of their cross sections. (**e**) Microscopic image of the cross-section of the connected coupling structures. (**f**) Schematic of the coupling wing model. Forewing and hindwings overlap and can be stored compactly. When the forewing pivots to the flight position, its coupling (yellow) on the trailing edge hooks the hindwing’s coupling (green), and they deploy together. The two wings are locked in flight position and behave as one while flapping. (**g**, **h**) Flapping drone equipped with coupling wings. Secondary veins are designed using the WCV algorithm.

Figure 3 illustrates the coupling-type wings inspired by the cicada wing. Cicada wings have groove-type coupling structures that interlock with each other from the top and bottom (Fig. 3a–e). The proposed method can only be built on the top side of the wings; therefore, we printed hindwings from the reverse side and turned them for assembly. The actual coupling structure in cicada wings has a complex cross-sectional shape with strong connections (Fig. 3e). Considering the ease of manufacturing with FDM, we used a simple hook-shaped coupling that can be fabricated without a support material (Fig. 3f). The wings were stacked and stored in a backward direction, similar to the actual cicada wings (Fig. 3g). When the forewing was rotated to the flight position, the hindwings were moved together by the coupling structures. Finally, the two wings were interlocked in the final position and formed a single wing (Fig. 3h). It was confirmed that this simple-shaped coupling can connect the wings during flight (Movie S4).

## Discussion

To understand the mechanics of insect flight, researchers have proposed a variety of models in a logical sequence, from conceptual models through simple physical models to full-scale numerical models^47^. The goal of the proposed method is to practise experimental approaches and bioinspired designs focusing on conceptual or physical models rather than to mimic real wings as accurately as possible. Therefore, the proposed 3D printed wings differ from real insect wings in many aspects, and it is important to understand the differences and advantages for future applications. Here, we would like to clarify the differences between real and 3D printed wings.

The proposed models are printed on flat films and are therefore basically two-dimensional, but actual insect wings include many 3D constructional elements. Typical examples are wing corrugations^48,49^, and basal complexes^50,51^, which are often studied in dragonfly wings. It is known that these three-dimensional structures not only increase the rigidity of the wings but also play a major role in automatic internal mechanisms that control wing twisting and the angle of attack during flapping cycles^48–51^. These effects are difficult to reproduce using a two-dimensional printed wing. A possible method to implement 3D constructional elements is to attach a film on a 3D mold with relief or corrugation and 3D print the veins on it.

We used simple WCV patterns with uniform cross-sectional beams as the secondary veins; however, actual secondary veins have a more complex distribution and form and are efficiently designed to act mechanically as 3D trusses or space frames. The next challenge is the automatic optimization of the secondary veins according to the stress path and required deformation by using the generative design and optimization techniques. It is also expected that such an advanced method will be useful for implementing the local areas and patches of flexibility and elasticity, or variations in the elastic moduli of the membrane found in real insect wings, which is not considered in the proposed models. These factors contribute to the flight mechanism by forming camber and controlling the angle of attack during up- and down-stroke cycles^47^. A combination of the venation-generating program with the optimization method that enables the design of the deformation of wings is capable of reproducing these advanced aerodynamic effects.

The veins are rigidly connected to each other in the proposed models, but it is known that dragonfly and damselfly wings contain elastically connected microjoints that efficiently cause twisting when flapping^52,53^. The nodus found on the leading edge of dragonfly wings are unidirectional hinges that provide higher resistance to aerodynamic forces during the downstroke^54^. These elastic hinges are relatively easy to implement in 3D printing and are expected to improve the flight performance of the proposed models.

The foldable wings in real insects have more than one elastically stable state, and the insects skilfully use elastic energy to achieve wing folding and unfolding^33,37,38^. Such elastic behavior was not considered in the proposed foldable wings. A method to design a bistable wing using a non-rigid foldable triangular pattern has been shown in previous studies^39^. This study used a groove-type joint for coupling-type wings. Another possible model is the hook-shaped coupling mechanism found in Hymenoptera^55^ or Hemiptera^56^. Bioinspired mechanical joints based on those of bees and wasps have been developed^57^. It is expected that the proposed coupling wings can be improved by incorporating these findings.

The Young’s modulus of the filament used for the vein is 1.9 GPa. The OPP film used for the membrane generally shows a Young's modulus of 1.7–3.9 GPa. These values are relatively close to those of real insect wings reported previously. For example, the elastic modulus of the hindwing membrane of the desert locust measured experimentally is 1.0–5.0 GPa^58^. Although there are few experimental datasets available for the Young’s modulus of wing veins, it is estimated to be approximately 3 GPa with reference to the physical characteristics of the cuticle of the leg^59^. In Ref.^60^, the natural frequencies of the wings of dragonflies, beetles, and cicadas, were determined using a laser displacement sensor. From the results, the frequency ratios (flapping frequency/natural frequency) are in the range of 0.35–0.43. From the results of our thrust tests, the frequency ratios of the prototype wings at a flapping frequency of 10 Hz, which shows the maximum thrust, are in the range of 0.5–0.714 and larger than that of actual insect wings. This difference can be attributed to the difference in rigidity and mass; that is, the actual wing has higher rigidity and lighter mass due to its three-dimensional structures, such as corrugation.

Considering the scalability, the possible size of the current setup is approximately 1.5–2 times larger than the real size of large insects. However, the Reynolds number of our thrust experiment was in the range of 3000–15,000. This means that these models can realize the same aerodynamic effects in bee or dragonfly sizes (Re = 1000), because the aerodynamic mechanism is almost unchanged at Re 1000–15,000. As long as FDM is used, the minimum diameter of a vein is constrained by the nozzle diameter (generally 0.2–0.4 mm). For miniaturization, it is necessary to consider using a different type of 3D printer, such as a stereolithography apparatus. Flapping drones have a lower speed at the end of the wing compared to rotor drones and are much safer to contact. Additionally, the PP wings used in this study are safer than a carbon frame, which is usually used for MAV wings because they do not scatter when broken, enabling the construction of devices that coexist in the real world.

Insect flights are highly interdisciplinary coupling programs that encompass different fields, including physics (i.e., structural mechanics, material mechanics, fluid mechanics, and aerodynamics) and biology (such as form, ecology, and evolution). Therefore, this problem cannot be solved in a single, closed research group. One promising approach is to consolidate knowledge in an open science framework and solve problems one by one throughout the academic community. The proposed prototypes have inferior flight performance compared to the previously reported flapping MAVs. However, the strength of the proposed method is its ease of reproduction, customization, and sharing; therefore, it is suitable as a platform for the above-mentioned open science approach. Sharing physical models that anyone can easily access facilitates communication between researchers in different fields. It is possible to sophisticate the models by repeating the all-at-once and rapid trials and errors on this open science platform. This is the same approach as evolution—the method that insects actually use to develop excellent abilities. The three proposed models can be used as the initial models of the evolutionary approach.

## Methods

### 3D printing process

Figure S1 shows a schematic of the insect wing 3D printing method. This study mainly used a large 3D printer (Bellulo 400, Systemcreate Co., Ltd., Osaka, Japan). A glass plate was added to the original print bed to fix the films. The membrane material used was oriented polypropylene (OPP) film envelopes (0.03 mm thick, ITOCHU Retail Link Corporation, Tokyo). The glass plate was enclosed in the OPP envelope, and the film was fastened by vacuuming air using a pump through a tube. The frames were built using a glass wool-filled PP filament (3D magic, NANODAX Co., Ltd., Tokyo) at 55 °C and 230 °C as the bed and extruder temperatures, respectively. We also used a desktop 3D printer (Lepton 2, MagnaRecta, Inc., Tokyo) for part-by-part printing. In the case where a 3D printer has a removable glass bed, it is possible to fix the film directory on the original bed using the above method. A detailed 3D printing process is presented in Movie S2. We confirmed that the PP film and PP filament show good adhesion properties when using this method; furthermore, the specimens (length: 80 mm, width: 12 mm, thickness: 2 mm) were 3D-printed on rectangular films, and the peeling forces were measured via 180° peel tests with a tension testing machine (MCT-2150, A&D Company, Ltd., Tokyo). The testing method and conditions were based on ISO 29862:2007 (self-adhesive tape–determination of peel adhesion properties). The results showed that the average adhesion strength is 5.5 N/cm, which is almost the same as that of the commercially used strong-type duct tapes and efficiently high for our purpose.

### Thrust test

The time-averaged thrust for each wing model was measured using a mechanical flapping machine. The machine with a DC motor and linkage oscillated the wing base in only the flapping direction (up and down strokes) with an amplitude of 10°. Due to the aerodynamic and inertial forces, bending and torsional deformation of the wing are passively generated during a flapping cycle. Figure S2a shows the schematic of the test setup. The test specimens were 3D-printed using the generated venation patterns. Figure S2b shows the specifications of the test wing (AB type). The margin frame used a linearly tapered shape with 3.0 to 0.5 mm thickness for efficiency of weight. The wing was fixed to a wing arm with a length of 40 mm so that the leading edge of the wing was directed downward. The thrust generated downward by the flapping wing was measured as a time-averaged value in 5.5 s with an electric balance (Shimadzu BX3200H, with a minimum resolution of 0.01 gf). The flapping frequency was set as 5.0–17.5 Hz with an increment of 2.5 Hz, which was measured with a laser displacement and fast Fourier transform analyzer. The thrust at each frequency was measured three times and averaged.

## Supplementary Information

Supplementary Video 1.

Supplementary Video 2.

Supplementary Video 3.

Supplementary Video 4.

Supplementary Information.

## Data Availability

All 3D models and codes shown in this study can be found in Ref.^46^.
