# Supplementary material for: Insect wing 3D printing
Source: Sci Rep. 2021 Oct 14;11:18631. doi: 10.1038/s41598-021-98242-y (PMC8516917; doi:10.1038/s41598-021-98242-y)
Supplement: Supplementary file 5 — Supplementary Information. [file 41598_2021_98242_MOESM5_ESM.pdf]

Supplementary Materials for

Insect wing 3D printing

Kazuya Saito[1]*, Hiroto Nagai[2], Kai Suto[3], Naoki Ogawa[4], Young ah Seong[5], Tomohiro Tachi[3], Ryuma Niiyama[5], Yoshihiro Kawahara[5]

[1]Kyushu University, Faculty of Design, Fukuoka, 815-8540, Japan
[2]Nagasaki University, Graduate School of Engineering, Nagasaki, 852-8131, Japan
[3]The University of Tokyo, Graduate School of Arts and Sciences, Tokyo, 153-8902, Japan
[4]Tokyo University of Agriculture, Kanagawa, 243-0034, Japan.
[5]The University of Tokyo, Graduate School of Information Science and Technology, 113-8654, Tokyo, Japan

*k-saito@design.kyushu-u.ac.jp

This PDF file includes:

Supplementary text
Figures S1 to S3
Legends for Movies S1 to S4

Other supplementary materials for this manuscript include the following:
Movies S1 to S4

Supplementary Text

SM text 1: WCV design system.

We developed a WCV design system using Rhino 6.0 and Grasshopper. In this system, users can interactively design the WCV venation pattern by modifying the input (Movie S1).
User inputs are as follows.

A. Closed poly-curves indicate the wing shape and primary veins defining the border of the WCV.
B. The numbers of the Voronoi cells in the primary vein of 1.
C. NURBS surface defining the density function of the weight.

The system processes the input data as follows. First, the system creates as many initial generator points as the number given in B in each closed poly-curve A, according to the density distribution given by NURBS surface C. Solid S_{strimed} is defined by extruding each Voronoi cell to make a prism, and by trimming with NURBS surface C. In this process, by setting equally distributed random points in the 3D space, the points existing in S_{strimed} are projected on the wing plane, and the initial generators with the objective density distribution (given by NURBS surface C) are obtained. Next, the Lloyd algorithm converts the pattern into a WCV vein pattern. The weighted centroid was calculated by projecting the centroid of each S_{strimed}. Using the NURBS surface as the interface makes it possible for users to design a proper density distribution intuitively, by operating the control points of the NURBS surface.

SM text 2: Design method for foldable wings.

Insects use various folding techniques according to their habits, body shape, and size. Among these, we focused on wing folding in relatively large beetles. Although the compactness of folded wings is inferior to that of small-sized beetles, such as ladybirds[38] and rove beetles[37], their wings have thick veins joined with clear articulations, which confer high stiffness and strength in wings and can be reproduced easily with artificial structures. Figures S3 a and b show the outline of the wing folding scheme in a horn beetle (*Trypoxylus dichotomus* (L. 1771)). The wings are supported by three main frames: mediocubital loop (MCL), radiomedial loop (RML), and anterior margin frame (AMF) (Fig. S3a and b). The leading edge (RML+AMF) has articulation (costal hinge), and the AMF rotates in the plane of the wing. These three frames are mechanically restricted with a triangular pattern found in the wing center, as shown in Figure S3c. The flat-foldable condition in this crease pattern is represented as follows (39).

$$\alpha_t + \beta_t = \pi \qquad (1)$$

$$\gamma_t + \delta_t = \pi \qquad (2)$$

$$\frac{\sin(p_1)}{\sin(m_1)}\frac{\sin(m_2)}{\sin(\beta_2)}\frac{\sin(\alpha_2)}{\sin(\gamma_3)}\frac{\sin(\beta_3)}{\sin(p_2)} = 1 \qquad (3)$$

The crease pattern of the 3D printed foldable wing is designed by defining length l and angles p_1, p_2, m_1, and m_2, and calculating the remaining parameters from Eq. (1–3). In the actual process, we used the generative design tool (Rhino and Grasshopper[44]), and made the code to check the folded/unfolded shape of wings at given design parameters (Movie S3)[46]. Figures S3d–g show the detailed design of the printed 3D model. The compliant hinge is printed as a 0.1 mm thick and 0.5 mm wide PP bridge. The main center facets are a reinforced PP plate (0.2 mm thick), and the crease pattern is implemented by 0.5 mm gaps on the fold lines. The rear end of the wing has a hook for fixing (blue part in Fig. S3f), which captures the buck end of the wing base part and deploys the MCL when the wing moves to the flight position, as shown in Fig. S3f. Figure S3g shows the actual crease pattern used. The detailed designing process and models are presented in Movie S3.
These models do not consider rigid foldability, because the reinforced facets allow elastic deformation at some level. To increase the rigidity of the wing, rigid foldability conditions should be considered.

$$u(\gamma_1, \delta_1)\, u(\alpha_2, \delta_2) \,/\, u(\beta_3, \alpha_3) \;-1 = 0 \qquad (4)$$

Here,

$$u(x, y) = \frac{1-\tan\left(\frac{x}{2}\right)\tan\left(\frac{y}{2}\right)}{1+\tan\left(\frac{x}{2}\right)\tan\left(\frac{y}{2}\right)} \qquad (5)$$

The value of the left side of Eq. (4) can be checked in the Grasshopper code (Movie S3), enabling the identification of rigid foldable crease patterns.

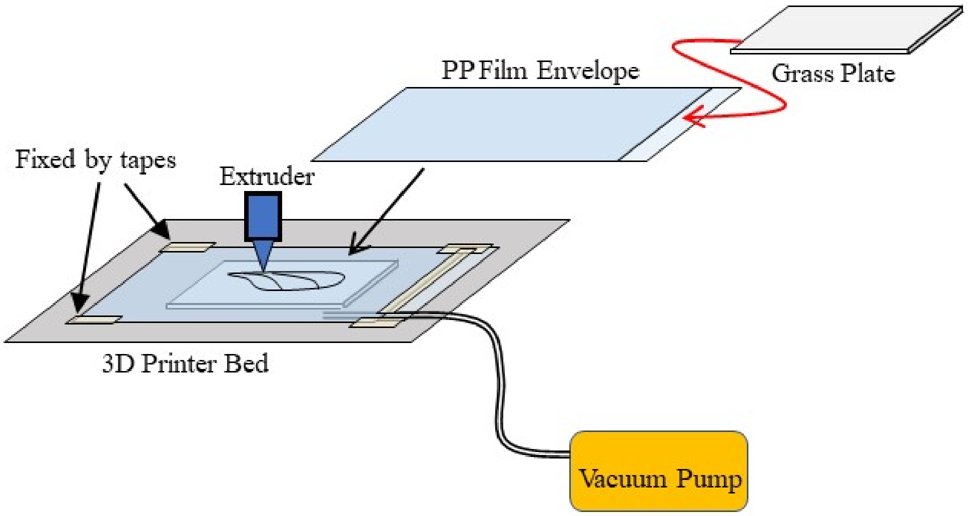

Figure S1. Schematic of insect wing 3D printing method. The OPP film envelope is tightly fastened on the surface of the glass plate by vacuuming the inner air via a pump. Tapes on the original 3D printer bed fix the glass plate with film. The vacuum pump keeps running during 3D printing.

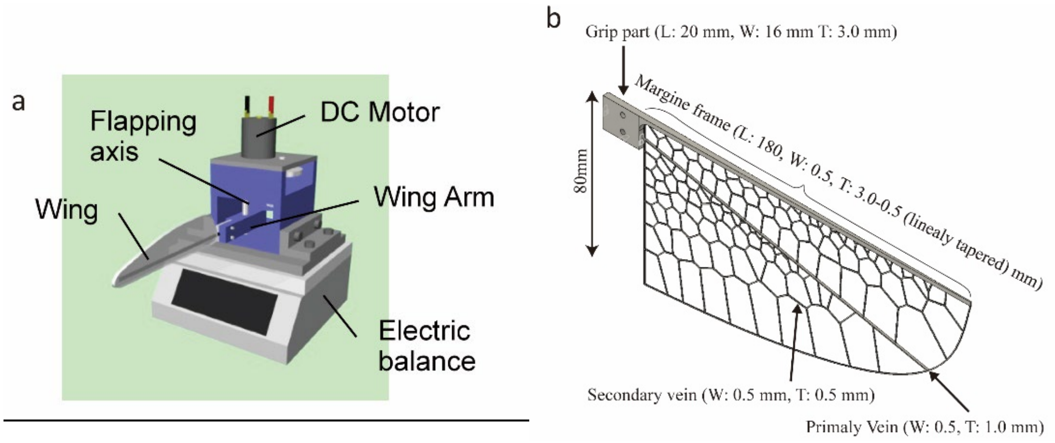

Figure S2. Thrust test for the printed wings. (a) Schematic of the experimental setup. (b) 3D model of the test wing (AB type).

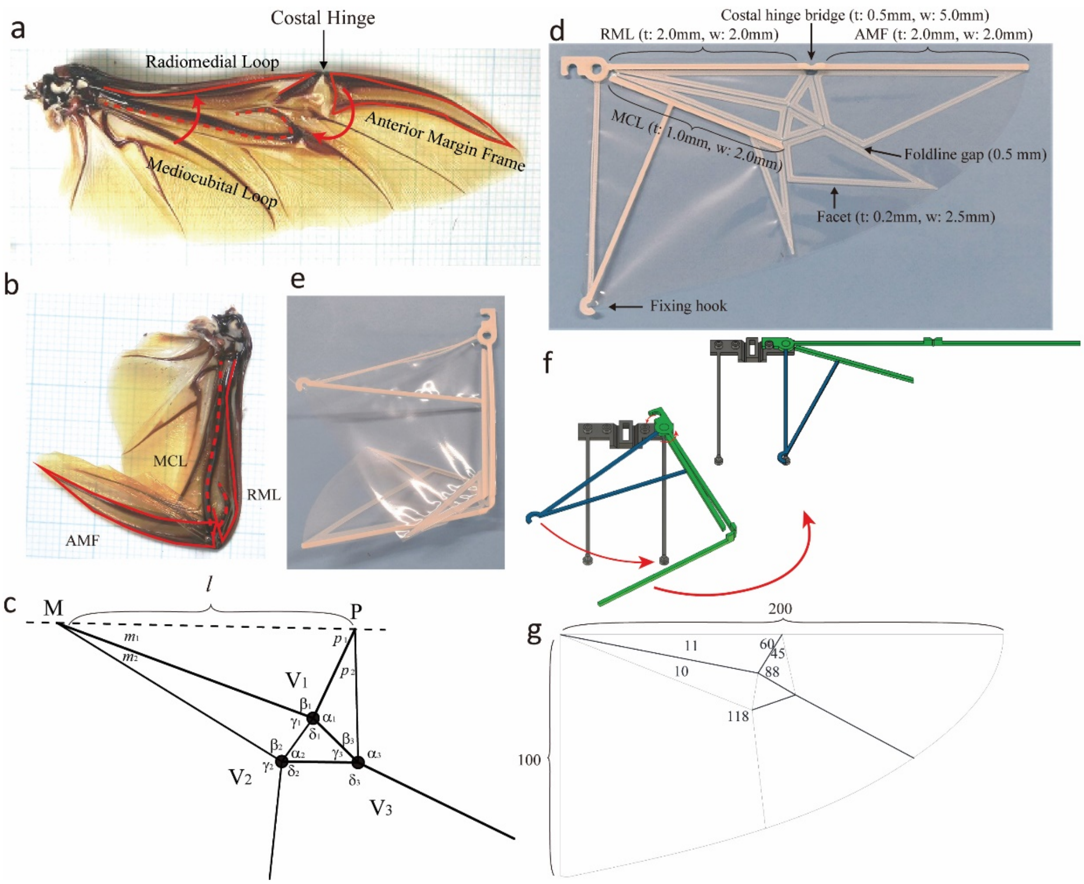

Figure S3. Schematic of the foldable wing. (a, b) Wing of Japanese horn beetle in unfolded and folded shape. Three frames, RML, MCL, and AMF, mainly support wing shape. RML and AMF are connected with the pin-joint-like articulation (costal hinge), which causes in-plane rotational movement on the AMF and achieves folding. This movement is caused by opening the RML and MCL on the wing base. (c) Triangular crease pattern found in typical beetle wings. This pattern is found at the wing center, and has the function to connect the movement among three main frames mechanically. (d) 3D printed foldable wing. Three main frames and facet reinforcing are printed on PP film. The costal hinge is made using the compliant hinge for simple manufacturing. (f) Wing deployment mechanism. The wings are stored backward with in a folded shape, and deployed by rotating around the base joint. Simultaneously, the hook is connected with the protrusion on the wing base; the MCL and RML are opened; and the AMF is extended because of the triangular pattern. (g) Angles and sizes of the wing crease pattern.

Movie S1 (separate file). Designing the WCV wing.

Movie S2 (separate file). 3D printing the WCV wing.

Movie S3 (separate file). Designing the foldable wing.

Movie S4 (separate file). Wing coupling motion in a cicada take-off and designing the coupling-type wing.
